# The whole genome sequence of Polish vaccine strain *Mycobacterium bovis* BCG Moreau

**DOI:** 10.1128/spectrum.04259-23

**Published:** 2024-05-17

**Authors:** Katarzyna Krysztopa-Grzybowska, Jakub Lach, Maciej Polak, Dominik Strapagiel, Jaroslaw Dziadek, Marcin Olszewski, Aleksandra A. Zasada, Aniela Darlińska, Anna Lutyńska, Ewa Augustynowicz-Kopeć

**Affiliations:** 1 Department of Sera and Vaccines Evaluation, National Institute of Public Health NIH – National Research Institute, Warsaw, Poland; 2 Biobank Lab, Department of Oncobiology and Epigenetics, Faculty of Biology and Environmental Protection, University of Lodz, Lodz, Poland; 3 Mycobacterium Genetics and Physiology Unit, Institute of Medical Biology, Polish Academy of Sciences, Lodz, Poland; 4 Chair of Drug and Cosmetics Biotechnology, Faculty of Chemistry, Warsaw University of Technology, Warsaw, Poland; 5 Department of Medical Biology, National Institute of Cardiology, Warsaw, Poland; 6 Department of Microbiology, National Tuberculosis and Lung Diseases Research Institute, Warsaw, Poland; CNRS-University of Toulouse, Toulouse, France

**Keywords:** BCG vaccine, BCG Moreau, BCG Moreau PL, tuberculosis, next-generation sequencing, complete genomic sequence

## Abstract

Currently, tuberculosis immunoprophylaxis is based solely on Bacillus Calmette-Guérin (BCG) vaccination, and some of the new potential tuberculosis vaccines are based on the BCG genome. Therefore, it is reasonable to analyze the genomes of individual BCG substrains. The aim of this study was the genetic characterization of the BCG-Moreau Polish (PL) strain used for the production of the BCG vaccine in Poland since 1955. Sequencing of different BCG lots showed that the strain was stable over a period of 59 years. As a result of comparison, BCG-Moreau PL with BCG-Moreau Rio de Janeiro (RDJ) 143 single nucleotide polymorphisms (SNPs) and 32 insertion/deletion mutations (INDELs) were identified. However, the verification of these mutations showed that the most significant were accumulated in the BCG-Moreau RDJ genome. The mutations unique to the Polish strain genome are 1 SNP and 2 INDEL. The strategy of combining short-read sequencing with long-read sequencing is currently the most optimal approach for sequencing bacterial genomes. With this approach, the only available genomic sequence of BCG-Moreau PL was obtained. This sequence will primarily be a reference point in the genetic control of the stability of the vaccine strain in the future. The results enrich knowledge about the microevolution and attenuation of the BCG vaccine substrains.

**IMPORTANCE:**

The whole genome sequence obtained is the only genomic sequence of the strain that has been used for vaccine production in Poland since 1955. Sequencing of different BCG lots showed that the strain was stable over a period of 59 years. The comprehensive genomic analysis performed not only enriches knowledge about the microevolution and attenuation of the BCG vaccine substrains but also enables the utilization of identified markers as a reference point in the genetic control and identity tests of the stability of the vaccine strain in the future.

## INTRODUCTION

Tuberculosis (TB), formerly known as the white plague, is an infectious disease caused by the *Mycobacterium tuberculosis* species complex. Because of its infectious nature, chronic progression, involving complex defense mechanisms of the immune system, the need for long-term and exhaustive treatment, the emergence of multidrug resistant forms, and TB-HIV coinfections, TB is a constant burden and public health challenge (1). TB has been one of the main causes of morbidity and human mortality in the world for centuries. According to World Health Organization (WHO), 10 million people worldwide fell ill with TB in 2019, with 1.4 million deaths. The disease affects people of all ages, while the greatest burden is observed among adult men (56%) compared to the group of adult women (32%) and children (12%). Almost 70% of all global TB cases occur in eight countries: India (26%), Indonesia (8.5%), China (8.4%), Philippines (6%), Pakistan (5.7%), Nigeria (4.4%), Bangladesh (3.6%), and South Africa (3.6%) (2). It is estimated that approximately 25% of the world’s population are carriers of *M. tuberculosis,* and the active form of the disease will affect 5%–10% of this population (3, 4). Approximately 8.2% of all TB cases worldwide in 2019 were people with HIV. The significant exacerbation of the epidemiological situation of TB in the world is mainly due to multidrug- or rifampicin-resistant TB (multidrug or rifampicin-resistant [MDR/RR]-TB), which caused 3.3% of new cases and 18% of previously treated patients in 2019. India (27%), China (14%), and the Russian Federation (8%) have the highest number of cases of MDR/RR-TB (2).

*Mycobacterium bovis* Bacillus Calmette-Guérin (BCG) is the only vaccine currently available against TB. It is a live-attenuated vaccine that is one of the oldest and most widely used vaccines in the history of medicine. About 100 million newborns around the world receive it every year. The original BCG strain was obtained by attenuation of the bovine TB pathogen *M. bovis* using passages in the years 1908–1921 (5). Since 1924, the parent strain of *M. bovis* BCG has been distributed to laboratories around the world to start vaccine production. This strain has been passaged and despite attempts to standardize this process, different conditions in individual laboratories led to the isolation of about 50 genetically separate substrains of BCG, for example, BCG-Pasteur, BCG-Tokyo, BCG-Danish, and BCG-Moreau (6, 7). Stopping further differentiation of the BCG strain became possible thanks to the development of a system of lyophilized seed lots in the 1960s and then issuing detailed recommendations by the WHO regarding the permitted number of passages in the BCG vaccine preparation process (5). The effectiveness of the BCG prophylactic vaccination estimated in many clinical and epidemiological studies varies. The greatest effectiveness of BCG vaccination was observed in North America and Northern Europe, while the lowest effectiveness was noted in tropical regions. The observed differences, as the vaccine researchers argue, may reflect differences between populations in terms of: exposure to environmental nontuberculous mycobacteria (NTM), BCG substrains and immunization schedules used, the virulence of *M. tuberculosis* and other factors, such as chronic intestinal parasitic infections, different concentrations of vitamin D, and iron of vaccinated persons (8–10).

Currently, most of the world’s population is supplied with BCG vaccines purchased by UNICEF on behalf of the Global Alliance for Vaccines and Immunization. UNICEF uses only four BCG vaccine suppliers that use three BCG substrains: BCG-Danish (Statens Serum Institute in Denmark), BCG-Russia (BB-NCIPD in Bulgaria and Serum Institute in India), and BCG-Tokyo (BCG Japan Laboratory). More than 90% of all BCG vaccines are produced globally from the BCG-Danish, BCG-Russia, BCG-Tokyo, BCG-Pasteur, and BCG-Moreau RDJ (11).

The BCG-Moreau strain is derived from the original BCG strain that was sent from the Pasteur Institute in Paris to Brazil by Dr. Julio Elvie Moreau on May 22, 1925. In 1951, Prof. Arlindo de Assis, Director of the BCG Center in Brazil, submitted the BCG-Moreau strain back to Paris for a comprehensive examination. The strain was donated to the Serum and Vaccines Factory in Lublin in 1954 by Dr. Van Deinse, the head of the BCG production laboratory at the Pasteur Institute. A year later, the production of the native BCG vaccine from the BCG-Moreau substrain began in Poland. From then on, the percentage of newborns vaccinate intradermally in Poland has been steadily increasing (12). Currently, more than 90% of newborns born in Poland are vaccinated with BCG each year (13).

The *M. bovis* BCG Moreau strain (for the purposes of the study, this strain will be marked PL) has not been genetically characterized so far, although it has been used for TB vaccine production in Poland for over 65 years. This study analyzes the whole genome sequence of *M. bovis* BCG Moreau PL and compares it with the genome of the closest BCG substrain (*M. bovis* BCG Moreau RDJ) and the most distant substrain (*M. bovis* BCG Pasteur). Additionally, five lots of the vaccine strain from 1957 to 2015 were compared to track the acquisition of mutations over time and to assess the genetic stability of the Polish strain.

## RESULTS

### General genomic features of BCG Moreau PL

**FIG 1:**
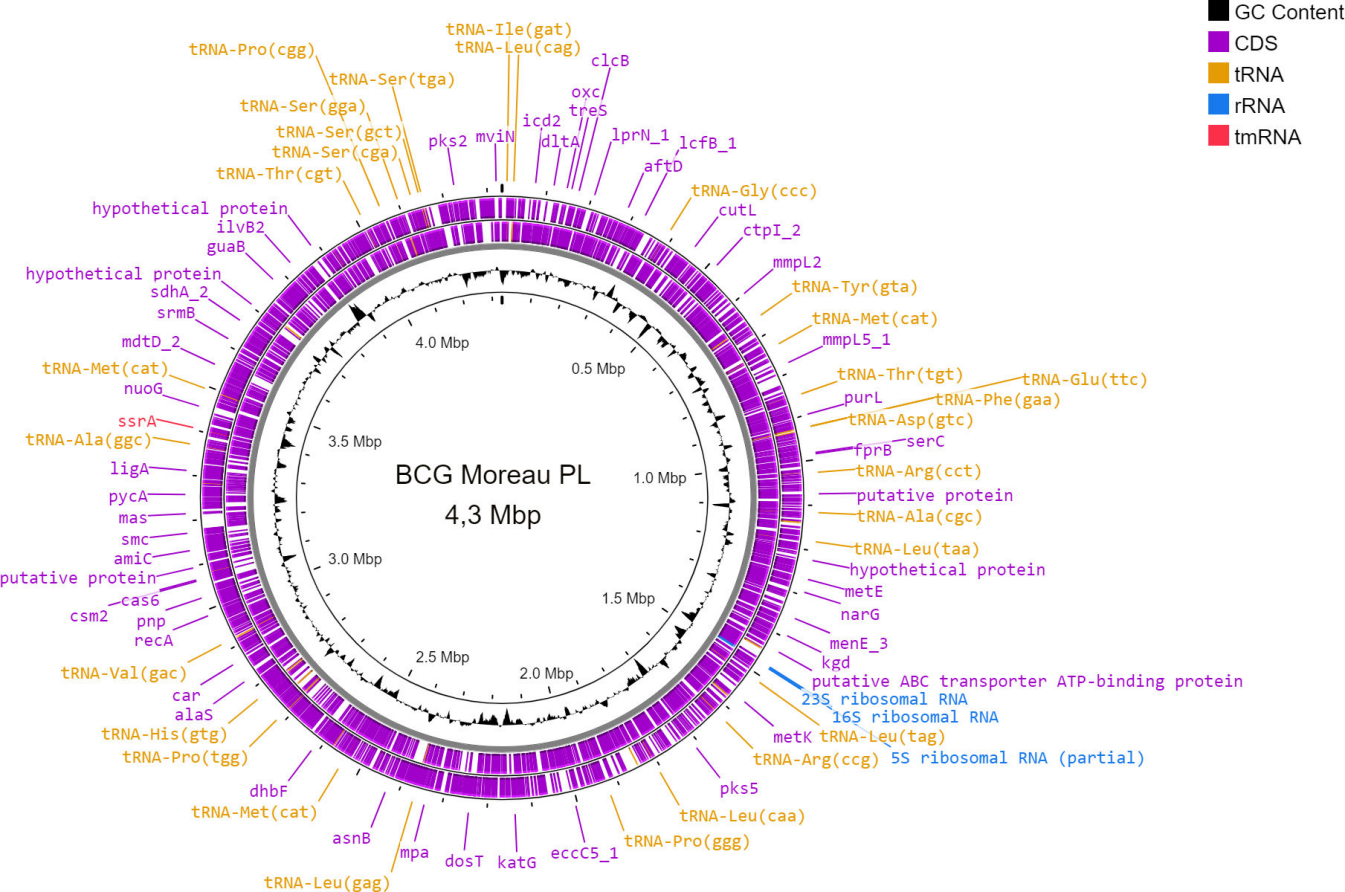
Circular representation of general genomic features of *M. bovis* BCG Moreau PL using Proksee (https://proksee.ca) (15). The scale is shown in megabases on the black central circle. The next circle shows the guanine-cytosine content (GC content). The two outer violet circles show forward and reverse strand coding DNA sequence (CDS), respectively. Some genes (violet), tRNA (orange), rRNA (light blue), and tmRNA (red) are pointed in the outer violet circle with the Proksee’s default parameters.

As a result of the hybrid assembled of long-reads obtained from third generation sequencing on the MinION platform and short-reads obtained from second generation sequencing on the Illumina platform, a preliminary genome sequence of the Polish vaccine strain was obtained. Carried out during the research, numerous verifications and corrections allowed to obtain the full sequence of *M. bovis* BCG Moreau PL, which was deposited in the GenBank database under the number CP085532. The completeness of this assembly estimated using the CheckM tool (14) was 99.78%, and the contamination was 0.6%. The *M. bovis* BCG Moreau PL comprises a circular chromosome of 4.3 Mbp, with an average G+C content of 65.65%. An automatic annotation carried out in accordance with NCBI Prokaryotic Genome Annotation Pipeline (PGAP) scheme showed 3,786 genes encoding for proteins, 3 genes for rRNA, 45 genes for tRNA, 3 ncRNA genes, and 222 pseudogenes (Fig. 1).

### Genetic variations in comparison to *M. bovis* BCG Moreau RDJ

**FIG 2:**
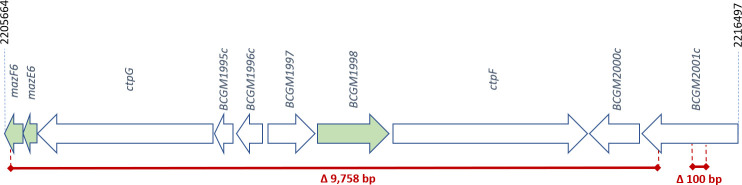
Schematic diagram of the two largest deletions identified in the *M. bovis* BCG Moreau PL genome relative to the *M. bovis* BCG Moreau RDJ reference genome. Genes associated with mycobacterial virulence are colored in green.

The results obtained confirmed the presence, in the BCG-Moreau PL genome, of the most characteristic changes of the BCG-Moreau strain described in the literature (16–19). However, there was no deletion of 876 bp in the gene homologous to *Rv3887c*, which confirms the results of our previous studies (20). Taking into account the comparative analysis of the *M. bovis* BCG Moreau PL and *M. bovis* BCG Moreau RDJ (AM412059.2) genomes, the presence of 175 mutations, including 143 single nucleotide polymorphism (SNP) mutations and 32 INDEL mutations was found in the Polish strain (Table S1a). Among the identified SNP mutations, 77 were located in the coding regions and caused changes in the amino acid sequences of the proteins, while three could have affected the encoded proteins due to their location in the probable promoter regions. Among non-synonymous point mutations, mutations in genes encoding Pro-Glu (PE) proteins accounted for more than half (45/77), of which 29 concern the *PE_PGRS43b* gene. Another group consisted of mutations in proteins involved in intermediary metabolism and respiration (12/77), of which seven were located in the gene encoding biotin sulfoxide reductase *(bisC*). Eight mutations occurred in genes of proteins with unidentified functionality (hypothetical proteins), three in genes of proteins involved in lipid metabolism, and three in the gene encoding the transposase. The fewest non-synonymous SNPs were identified in the genes of regulatory proteins (2/77) and genes of proteins associated with the bacterial cell wall and cell processes (2/77). No single mutation of this type was identified among the genes associated with virulence, detoxification, and adaptation. The predominant number of SNP mutations in PE protein genes is indicative of their particular high polymorphism, occurring even in very closely related strains. The functional annotation of the identified SNPs using PROVEAN (21) showed that among the identified mutations, only one was potentially significant from the point of view of protein functionality. Among the INDEL mutations identified, 17 involved coding regions. The two largest deletions were 9,758 bp and 100 bp long (Fig. 2). These closely spaced deletions involved a total of nine genes. An in-depth analysis of genes that were completely or partially deleted in the *M. bovis* BCG Moreau PL genome, based on data available in the Mycobrowser database (mycobrowser.epfl.ch.) (22), showed the presence of 10 genes in this region, including 3 of them related to the virulence of mycobacteria: *mazF6* toxin gene, *mazE6* antitoxin gene and gene encoding one of the universal stress proteins. This region also contains genes encoding two of the twelve P-type ATPases found in the mycobacterial genome (CtpG and CtpF) and an important transcription factor (CmtR).

Analysis of the impact of these two deletions (9,758 bp and 100 bp) showed that eight whole genes: *mazE6*, *ctpG*, *BCGM1995c*, *BCGM1996c (cmtR*), *BCGM1997*, *BCGM1998*, *ctpF* and *BCGM2000c*, and fragments of the *mazF6* and *BCGM2001c* genes are removed. As per the *in silico* translation (insilico.ehu.es/translate) (23), the potential presence of two hypothetical proteins, with a length of 151 aa and 59 aa, was identified. Possible protein sequences were analyzed using the Domain Enhanced Lookup Time Accelerated BLAST (DELTA-BLAST) (24) algorithm for the non-redundant protein sequences database. The result was obtained only for the larger protein (151 aa) – it was 98% similar to a conserved *M. tuberculosis* membrane protein of uncharacterized function. The assembly of the sequence of this protein with the *BCGM2001c* gene product in Standard Protein BLAST showed the identity of the first 145 aa. The deletions described above led to the shortening of the protein in BCG-Moreau PL, but as modeling in SWISS-MODEL (25) showed, it still has 4 of the 12 transmembrane segments. The template modeling score (TM-score) calculated in the TM-align for the truncated protein relative to the reference was 0.33046 (26).

The TM-score was calculated for 37 proteins with significant SNP and/or INDEL mutations (26). In five cases (*PE_PGRS19*, *PE_PGRS54*, *PPE24*, *BCGM2001c*, and *BCGM3906c*), the identified mutations resulted in a change in protein folding.

### Genetic variations in comparison to *M. bovis* BCG Pasteur

Comparative analyses of *M. bovis* BCG Moreau PL and *M. bovis* BCG Pasteur (AM408590.1), 2 of the most genetically distant BCG substrains, identified a total of 69 significant mutations (non-synonymous SNPs and INDELs in CDSs or probable promotor regions), including 41 SNPs and 28 INDELs (Table S1b). The largest number of SNP mutations (14/41) affected genes encoding PE and Pro-Pro-Glu (PPE) proteins, of which eight were located in the *PE_PGRS28* gene. A total of 40 SNP mutations and 24 INDEL mutations common to *M. bovis* BCG Moreau PL and *M. bovis* BCG Moreau RDJ, which distinguished them from the *M. bovis* BCG Pasteur strain, were identified (Table S1a; Table 1). Many of them are important genetic markers characteristic of the BCG-Moreau substrain described in the literature (16–19). In the Polish BCG genome SNP D322G in the *phoR* gene, the lack of DU1, insertion of IS*6110* in front of the *phoP* gene, N-RD18, RD14, and RD2, deletion of the C-terminal end of the *fadD2* gene and the N-end of the *ppsA* gene, DU2-I, deletion of RD16, and deletion of S-RD13 were detected (Table S1b). Analysis of significant mutations differentiating the Polish BCG strain from the RDJ strain showed that 77/80 sequences with SNPs and 15/19 sequences with INDELs were identical to sequences in the *M. bovis* BCG Pasteur genome, indicating that most of the SNPs and INDELs differentiating *M. bovis* BCG Moreau RDJ and *M. bovis* BCG Moreau PL were accumulated in BCG-Moreau RDJ after the transfer of BCG-Moreau to Poland.

**TABLE 1:** The number of significant mutations identified in the *M. bovis* BCG Moreau PL genome compared to *M. bovis* BCG Moreau RDJ and *M. bovis* BCG Pasteur

Type	PL vs. RDJ	PL vs. Pasteur	PL vs. RDJ & Pasteur	PL & Pasteur vs. RDJ	PL & RDJ vs. Pasteur
SNP	80 (77, 3)^*a*^	41 (38, 3)^*a*^	1	77	40
INDEL	19 (17, 2)^*a*^	28 (26, 2)^*a*^	4	15	24

^*a*^ Mutations changing the amino acid sequence of a protein, mutations in the promoter region.

### Unique genome mutations of *M. bovis* strain BCG Moreau PL

**FIG 3:**
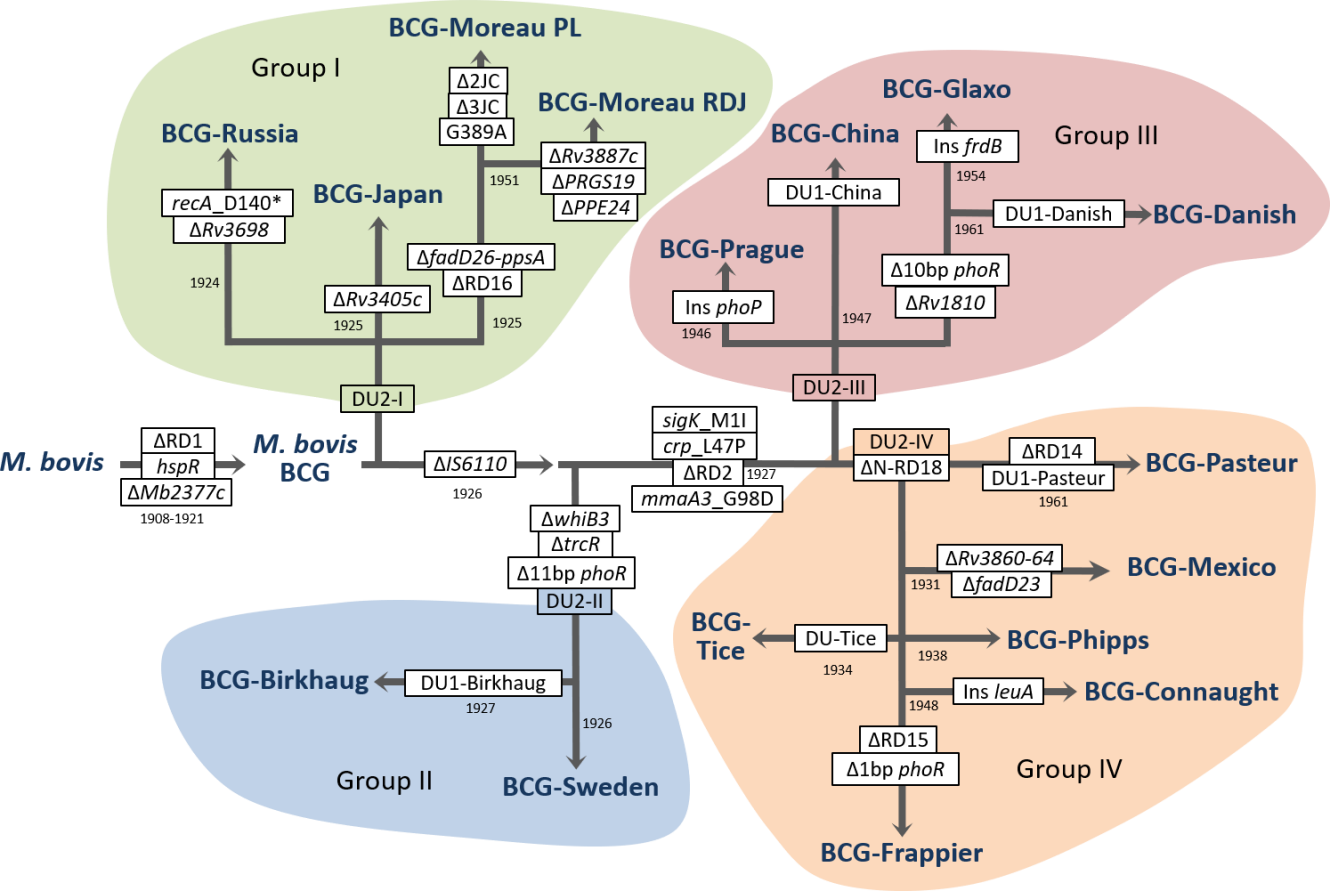
Diagram of the BCG microevolution, including the year a given substrain was obtained and the most important genetic changes (16–18, 27–29).

Of the five mutations (one SNP and four INDELs) that distinguished the Polish strain from both BCG-Moreau RDJ and BCG Pasteur (Table 1; Table S1b), there were potentially unique markers. Comparison of these regions with all available genomic sequences of BCG substrains deposited in GenBank revealed that the G389A SNP in the gene homologous to *Rv2553c* encoding one of the conserved membrane proteins and two INDELs: ∆9,758 bp (∆2JC) and ∆100 bp (∆3JC) are characteristic only of the BCG-Moreau PL genome (Fig. 3). The remaining two INDELs, described in Table S1b with no. 25. and 28. have also been identified in the following genomes: BCG-Danish (CP039850.1), BCG-Tokyo (AP010918.1), BCG-Russia (CP009243.1), and BCG-Sofia (CP064405.1).

### Genetic stability of the BCG-Moreau PL strain

In the study of the genetic stability of the *M. bovis* BCG Moreau PL strain, the results for five additional genomes of lots from different years obtained by next-generation sequencing (NGS) on the Illumina platform were analyzed. Genetic similarity analysis did not show any significant differences between lots. The average nucleotide identity (ANI) of the studied genomes was ≥99.988%, showing a very high similarity between them. Furthermore, all identified mutations were found to be common to the five lots tested.

## DISCUSSION

BCG–live, attenuated vaccine is the only vaccine used in immunoprophylaxis of TB since 1921. The specific conditions of the BCG vaccine production technology in different countries over the decades have caused specific genetic changes of the original BCG vaccine strain and the emergence of genetically diverse progeny strains, which have been referred to as BCG substrains. The BCG vaccines produced from different substrains are characterized by different immunogenicity, efficacy, and safety profile (6, 7, 18, 30–32). Although a hundred years have passed since the BCG vaccine was first used, despite the enormous progress in scientific research, it has not been replaced with a new preparation (33). Therefore, it seems that further work on a better characterization of the BCG vaccine strains will allow for a more reliable assessment of the effectiveness and quality control of vaccines, as well as a complete understanding of the mechanisms of action (11).

Taking into account the evidence that the genome of mycobacteria is susceptible to rearrangements, such as deletions or duplications, the genotyping of BCG substrains plays an important role in controlling the production processes in terms of the stability of the vaccine strains (11, 34). This is important because, in addition to genetic differences between BCG substrains, variants of the same substrain have also been observed. Well-documented examples of such variants include the BCG-Tokyo, BCG-Danish, or BCG-Pasteur substrains (17, 18, 27, 35–37). Given that the genetic variations of the vaccine strains may affect the effectiveness and safety of the vaccines produced, it is necessary to fully characterize individual BCG substrains and monitor their homogeneity over the years. Genetic stability studies have so far been mainly based on typing the multiple deletion (RD) and tandem duplication (DU) regions, that is, defined genetic markers that distinguish the substrains of BCG (20, 38–40). Methods based on the amplification of the genetic markers specific to particular BCG substrains defined in the literature are very simple, fast, and inexpensive, which makes them suitable for ongoing control of the of vaccine strain identity (41). However, these methods do not allow for the complete characterization of a strain, but only its selected regions. In recent years, the development of NGS techniques has enabled the precise identification of differences between some BCG substrains and the verification of their genetic stability (42). Asadian *et al.* (42) analyzed strains obtained from different batches of vaccines produced in the years 2010–2019 from the BCG-Pasteur substrain. Sequencing using the Illumina platform did not show batch-to-batch differences, yet only minor changes from the *M. bovis* BCG Pasteur 1173P2 reference genome were shown. The authors emphasized that it would be reasonable to support the sequencing-based technique with a short-read using a long-read technique to elucidate the sequence of regions containing repeats (42). Monitoring of spontaneous mutations in the whole genome has become possible with the development of methods based on NGS technologies and may turn out to be a new standard for the quality control of the BCG vaccine (43). Stability studies using NGS on Roche’s 454 platform were performed for the BCG-Russia strain. This study compared the results obtained for three seed lots: 311 (1963), 977 (1982), and 368 (2006). Only two low-grade lesions were identified, confirming the genetic stability of the BCG Russia substrain vaccine used in production (44). The latest genomic studies of the Russian working seed lot no. 368 of the BCG-Russia strain showed divergent results, including those with respect to the sequence of the *glnD* gene. The authors concluded that these data may suggest internal polymorphism between lots produced by different manufacturers (Medgamal and Microgen) resulting from differences in the production process. The observed heterogeneity may also be partly related to different bioinformatics tools and accepted qualitative criteria used in the analysis of NGS data, which was noted out by researchers (45).

**FIG 4:**
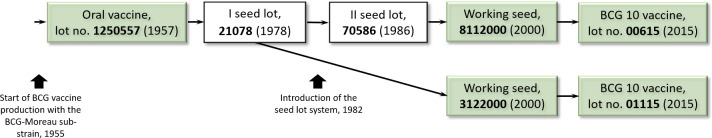
Diagram of the production of TB vaccines in Poland. The material used for the tests is marked in green.

In the years 1947–1950, mass vaccinations of children and adolescents aged 1–18 were carried out in Poland, using a vaccine based on the Danish substrain. The vaccination campaign was hampered by numerous postvaccination reactions in the form of lymphadenitis in newborns vaccinated orally. Replacing the route of administration changed the location of the reactions but did not reduce them and, in fact, increased their number. A significant improvement occurred in 1954 after the vaccine substrain was changed from Danish to Brazilian (BCG-Moreau), with virtually no complications. The BCG Moreau substrain is derived from the original BCG strain sent from the Pasteur Institute in Paris to Brazil by Dr. Julio Elvie Moreau on 22 May 1925. It was transferred to Poland in 1954 to the Serum and Vaccine Factory in Lublin. A year later, the production of the native BCG vaccine from the BCG-Moreau substrain began in Poland. The freeze-drying of strains was used in the production process from the beginning (12). The first vaccines made in Lublin were oral vaccines, produced since 1957 from lot no. 1250557, which was replaced after 82 passages with lot no. 21078, initially called the experimental seed lot, and then, after implementing the principles of the seed lot system in production in 1982, it was officially the first seed lot of the BCG vaccine in Poland (Fig. 4). After its exhaustion in 1986, the second seed lot no. 70586 was introduced. Currently, in the production of the “BCG 10 Anti-TB Vaccine” in Poland, Biomed-Lublin uses two working seed lots in parallel: working seed lot no. 8112000, derived from the second seed lot no. 70586 and working seed lot no. 3122000, derived from the first seed lot no. 21078. In this study, to test the stability of the Polish vaccine strain, NGS was carried out on the Illumina platform involving five lots from different years: oral vaccine no. 1250557 (1957), two current working seed lots no. 8112000 (2000) and 3122000 (2000), and final products no. 00615 (2015) and no. 01115 (2015).

This material selection was intended to identify possible genetic changes before and after the introduction of the seed lot system, differences between the working lots used for the production of the BCG vaccine and differences between a given working lot and the final product obtained from it. Analysis of genetic similarity of the five test lots of the *M. bovis* BCG Moreau PL strain did not show significant differences between lots. The average similarity of the nucleotide sequences of the working genomes obtained for each batch with the other four was ≥99.988%, which confirmed a very high similarity between them. Mutations and weak/null coverage identified for each lot were also analyzed based on Illumina sequencing data against the *M. bovis* BCG Moreau RDJ reference substrain genome. All identified changes were found to be common to the five batches tested. These results demonstrate the genetic stability of the *M. bovis* BCG Moreau PL strain over a period of almost 60 years, which is consistent with our own previous research on the genotyping of the Polish BCG lots (20). Therefore, it can be concluded that the manner, in which the vaccine strain was stored and passaged in Poland did not lead to genetic changes, and the identified mutations present in all five BCG lots occurred before 1957. However, it cannot be determined whether these changes occurred in Poland after receiving the BCG-Moreau substrain between 1954 and 1957, because we do not have the 1954 lot. The original BCG-Moreau strain that was sent from the Pasteur Institute to Brazil is also unavailable. Therefore, the data obtained in the study were compared to the genomic sequence of *M. bovis* BCG Moreau RDJ deposited in the GenBank database (19). The strain, sequenced by Gomes et al*.*, is a strain derived from the BCG vaccine currently used in Brazil and, therefore, probably different from the original BCG-Moreau strain, imported to Brazil in 1925, about 85 years earlier. It is worth to emphasize how important it is to know the full sequence of the strain used to produce the BCG vaccine administered to the entire population. In future studies, the Polish strain will constitute a reference point for comparing the sequences of strains isolated from patients with BCG-itis after a locally produced vaccine in order to verify whether genetic changes occurred *in vivo*. The stability analysis of BCG-Moreau PL showed that the markers we identified have not changed over almost 60 years. We can confidently suspect that this will not change because knowledge about the production of the BCG vaccine and maintaining quality standards, including the use of a freeze-dried seed lots system, has achieved enormous progress since the 1960s. However, with the full genome sequence now available, stability can be easily monitored and any changes detected.

In order to obtain the complete genome sequence of the Polish vaccine strain *M. bovis* BCG Moreau PL, two new generation sequencing technologies were used – Illumina (second generation) and MinION (third generation). The strategy of combining short-read sequencing, for example, NGS Illumina, with long-read sequencing, for example, NGS MinION, provides the most optimal approach to sequencing bacterial genomes and allows the identification of both small point mutations and major genomic rearrangements. As shown in the paper, hybrid *de novo* assembly, in which long NGS MinION reads form a “scaffolding” supplemented by short NGS Illumina reads, allowed to obtain a good quality single working genome sequence that did not require many corrections. This approach is supported by recent sequencing of the BCG-Danish 1331 strain (27). The authors pointed out that using short-read sequencing for genomes containing multiple repeat regions and large deletions and duplications can generate errors. Such sites can be effectively verified using long-read technology. That was the first published hybrid approach to genome sequencing of a BCG strain that identified inconsistencies in previous papers describing the sequence of the BCG-Danish substrain. The scientists also emphasized that the methodology, combining the two NGS technologies, is both time- and financially advantageous compared to the methods used in the past (27, 30).

Analysis of the study results confirmed the presence of characteristic changes of the BCG-Moreau strain described in the literature in the genome of the Polish strain BCG-Moreau PL (16–19), that is, lack of DU1 duplication, presence of DU2-I duplication, IS*6110* insertion before the gene *phoP*, C-terminal deletion of the *fadD2* gene and N-terminal deletion of the *ppsA* gene, RD16 deletion, S-RD13 deletion, and lack of deletion: N-RD18, RD14, and RD2. When analyzing the sequences containing the identified SNP mutations and INDEL mutations in the *M. bovis* BCG Moreau PL substrain genome against *M. bovis* BCG Moreau RDJ, the vast majority were shown to be identical to the sequences in the *M. bovis* BCG Pasteur genome. Therefore, these mutations probably occurred in the genome of *M. bovis* BCG Moreau RDJ and not in *M. bovis* BCG Moreau PL. However, mutations that distinguish the genome of the *M. bovis* BCG Moreau PL strain from the BCG genomes available in GenBank are one SNP mutation (G389A) in the gene homologous to *Rv2553c,* encoding conserved membrane protein and two INDEL mutations (∆9,758 bp and ∆100 bp). These deletions cover 10 genes, including among others: a toxin gene (*mazF6*), an antitoxin gene (*mazE6*), a gene encoding one of the universal stress proteins, two genes encoding ATPases (*ctpG, ctpF*), and a transcription factor (*cmtR*). All the genes mentioned are involved in the response to stress conditions and the survival of mycobacteria in the host tissues, although the mechanism of their action is different. The presence of free toxin MazF6 causes reprogramming transcription toward adaptation to stress conditions (46) while P-type ATPases, CtpG and CtpF, are critically important for the survival of mycobacteria because they are responsible for the detoxification of metal ions (Zn2+ and Ca2+, respectively) inside human macrophages during infection (47, 48). These changes may constitute one of the factors contributing to the higher safety of the Polish BCG vaccine compared with BCG-Moreau RDJ described in the literature (49). However, the exact significance of the mutations unique to the Polish strain, both in terms of their impact on the vaccine safety and immunogenicity, requires more detailed analyses in the future. In the next stage of the research, analogous comparisons at the transcriptome and proteome level to reference strains were planned. It will be intriguing to verify what alternative pathways of response to stress conditions have been developed in BCG-Moreau PL strain.

In the mid-1990s, intensive research began on the genetic diversity of BCG substrains. RD, tandem duplications (DU), and SNPs were identified. On the basis of the detected genetic markers differentiating the substrains and historical knowledge of their distribution, phylogenetic trees were constructed to present the microevolution of BCG substrains (17, 18, 50). In routine BCG vaccine identity testing, multiplex PCR is used, which allows BCG to be identified accurately to the substrain (41). However, it does not allow the differentiation of variants of the same substrain as BCG-Moreau RDJ and PL. The markers we identified that are unique to the Polish substrain allowed us to distinguish it from other substrains, including RDJ. Simple PCR using the 2JC primers we designed in the study gives product only for BCG-Moreau PL (Table S2).

Despite its imperfections, BCG has remained the only licensed vaccine against TB for over 100 years. Since 1974, it has been included in the WHO Extended Vaccination Program. The legitimacy of conducting BCG vaccinations in the world is primarily owned to the proven protection of children against the most severe forms of TB, meningitis, and miliary form. However, protection against the most common form – pulmonary TB – varies greatly between 0% and 80% (8). Randomized follow-up studies showed that no prior infection with *M. tuberculosis* or no exposure to environmental mycobacteria is associated with higher efficacy of BCG against pulmonary TB and possibly miliary and meningeal TB (51). However, it seems that many more factors are causing such a significant variation in vaccine effectiveness. One of the most important factors may be the BCG substrain used for its production (18). Ritz et al*.* in 2008 provided a critical summary of the available studies comparing the efficacy of BCG vaccines in animals and humans. The analysis confirmed that the induced immune response and protection against TB differed according to the BCG substrain used to produce the vaccine. However, the results were so contradictory that it was not possible to identify a single favorite among the BCG substrains. As emphasized, identification of the BCG substrain with the best protective properties would have a huge impact on TB control at the population level (52). Two large randomized clinical trials of BCG revaccination in Brazil and Malawi failed to demonstrate efficacy against TB, although both trials did not take into account *M. tuberculosis* infection status among participants (53–55). More recent studies in the uninfected population have shown the potential of BCG revaccination to protect against latent infection. Studies verifying these results, conducted on a larger cohort, are to be completed in 2026 (55, 56). This demonstrates a potential new use for BCG. However, not only revaccination of a specific target group but also changing the route of administration (57) and genome recombinations (58) are tested, which shows that BCG can still be the subject of promising research. The Polish strain, apart from the genetic markers characteristic of the Moreau substrain described in the literature, is a natural mutant in several genes related to the response to stress and intracellular survival in host cells. Among them are genes encoding two P-type ATPases, CtpG and CtpF. As a recent research has demonstrated, their deletion disturbs detoxification systems reducing mycobacterial virulence. The *ctpF* gene shows high level of activation under different conditions, including infection, oxidative/nitrosative stress and hypoxia, promoting a non-replicating persistence (NRP) or dormancy state of bacilli. The authors suggested that this may be a good target for the attenuation of *M. tuberculosis* in terms of obtaining a new vaccine candidate against TB (47). The advantages of whole cell live vaccines include a broad antigenic repertoire, triggering a diverse immune response, mimicking immunity elicited by natural TB infection (56). However, it is necessary to develop immunological correlates of protection, in the form of a set of biomarkers (biosignature), to facilitate the development and comparison of vaccines (59).

Not a single genetic marker, but all changes in the BCG substrain genome contribute to vaccine effectiveness in a given population. Therefore, it is reasonable to track these changes and characterize BCG genomes against other substrains and genomes of virulent strains of *M. tuberculosis*. This study found that among the significant non-synonymous SNP mutations identified in the *M. bovis* BCG Moreau PL genome, in relation to the *M. bovis* BCG Moreau RDJ genome and to the *M. bovis* BCG Pasteur genome, most mutations occurred in genes encoding the PE and PPE proteins, which proves their high polymorphism within the BCG substrains. This conclusion is consistent with scientific reports showing both sequence and expression level variability of the *pe*/*ppe* genes among BCG substrains (18, 60). PE and PPE are two large families of proteins specific for mycobacteria, whose genes occupy approximately 7% of the coding regions in the genome of the *M. tuberculosis* complex species. Their name comes from the conserved proline and glutamic acid motifs PE and PPE at the N-terminal ends of the proteins. The *pe*/*ppe* genes are of great interest to scientists due to their high variability (61). Although discovered more than 20 years ago, the function of these proteins, and the polymorphic subfamilies, in particular, remains unclear. Many studies suggest that some PE and PPE proteins play an important role in the virulence of mycobacteria (61–67). Genes encoding some of the PE and PPE proteins have been shown to be overexpressed for mycobacteria isolated from lung granulomas compared to bacteria cultured *in vitro* (63, 68). Surface exposure or secretion into the extracellular environment allows the PE/PPE proteins to interact directly with host cells. Some proteins probably activate signal transduction and modulate cytokine production via the TLR2/4 receptors on the surface of macrophages, others inhibit autophagy and inflammatory responses to avoid an attack by the immune system or shift the response from Th1 to Th2, which promotes intracellular survival of mycobacteria (69). This variation may result in different levels of protection for BCG vaccines produced from different substrains (60). In contrast, it is worth noting that BCG has a defect in the secretion of PE/PPE proteins due to the loss of the RD5 region, making it difficult to relate the results of research on virulent strains to vaccine strains in this matter (70, 71).

## MATERIALS AND METHODS

### Vaccine strains and DNA isolation

The genetic stability study analyzed the NGS results on the Illumina platform for five strain lots of the Polish *M. bovis* BCG Moreau strain derived from different years (Fig. 4). The oldest of the tested lots, no. 1250557, came from an oral vaccine that was freeze dried in 1957, 2 years after the start of production of the BCG vaccine in Poland from the Moreau substrain. Two more lots: no. 8112000 and no. 3122000, were produced from two different master seed lots in 2000. Both are working seed lots used currently to produce the Polish national BCG vaccine. The last two lots tested: no. 00615 and no. 01115 are the final products, that is, “BCG 10 Anti-TB Vaccine” produced in 2015, from different working seed lots 8112000 and 3122000, respectively.

The mycobacteria were cultured on Ogawa medium for 21 days at 37°C. Bacterial DNA was isolated according to the methodology of van Soolingen et al. 1991 (72) with minor modifications.

### Whole genome sequencing and reference genome

The DNA of the five tested lots of the *M. bovis* BCG Moreau PL strain was sequenced on the Illumina NextSeq 500 platform in a 2 × 250 bp configuration, using the NEXtera XT kit (Illumina) for libraries preparation (240× coverage per genome).

Genome sequencing of the working seed lot no. 3122000 of the *M. bovis* BCG Moreau PL strain was additionally carried out on the MinION platform (40× coverage). The libraries have been prepared using the Ligation Sequencing Kit (Oxford Nanopore Technologies).

### Genome assembly and analysis

In the first step, raw fastq files from the Illumina platform were trimmed using Trim Galore! to purify reads from the outermost sections and to remove poor quality sequences (73). Reads from the NGS Illumina platform for the five strain lots of the Polish *M. bovis* BCG Moreau substrain were aligned separately to the *M. bovis* BCG Moreau RDJ reference genome (AM412059.2) (19) using the Bowtie2 tool (74). Subsequently, consensus sequences were created for the five lots of the Polish BCG strain in BCFtools (75). In fastANI, the average nucleotide sequence similarity (ANI) was calculated for each lot in relation to the other four lots (76). Sequencing results obtained from the Illumina platform were used for the identification of mutations (variants) using breseq in the genomes of the five lots of the Polish BCG strain against the reference genome *M. bovis* BCG Moreau RDJ (77). The variant designation threshold was the occurrence of changes in reads with a frequency of at least 80%. The breseq program was also used to identify sites in the reference genome that were not covered by Illumina reads, which may indicate a deletion of a given region. All identified mutations detected by breseq, differentiating the five lots of the Polish BCG strain and the uncovered regions, were verified in Integrative Genomics Viewer (IGV) (78).

Reads from the MinION platform for the working seed lot no. 3122000 were aligned to the *M. bovis* BCG Moreau RDJ reference genome using the minimap2 tool (79).

The initial sequence of the Polish vaccine strain (working seed lot no. 3122000) was generated as a result of the hybrid genome assembly of long reads obtained from the MinION platform and short reads obtained from the Illumina platform using hybridSPAdes (80). All-important mutations, that is, those lying at CDS and at probable promotor regions (up to 50 nt upstream of the START codon), were verified by Sanger sequencing. The Sanger sequencing was performed using primers listed in Table S2.

Verifications and corrections allowed to obtain the complete sequence of the Polish strain *M. bovis* BCG Moreau (working seed lot no. 3122000), which was deposited in the GenBank database where it was automatically annotated with the “Best-placed reference protein set; GeneMarkS-2+” method according to the “NCBI PGAP” scheme and received accession number CP085532. Genome visualization was performed using Proksee (v6.0.2) (15).

In order to check in which genome of BCG Moreau the identified mutations occurred, the sequence of the Polish substrain was additionally compared with the most distant sister substrain of BCG – and *M. bovis* BCG Pasteur 1173P2 (AM408590.1) (18).

Among the significant mutations (non-synonymous SNPs and INDELs in CDSs or in probable promotor regions) identified in the *M. bovis* BCG Moreau PL genome, mutations specific only to the Polish vaccine strain were searched. For this purpose, sequences containing mutations from the BCG Moreau PL genome against both BCG Moreau RDJ and BCG Pasteur were compared with genomic sequences of the BCG substrains (taxid: 33892) deposited in GenBank using BLASTN (NCBI).

The theoretical impact of identified mutations was analyzed using the insilico.ehu.es tool (genetic code: Bacterial and Plant Plasmid) (23), and the functional category of proteins, in which mutations were identified was assigned according to mycobrowser.epfl.ch (22). Protein modeling was performed using SWISS-MODEL (25), and the impact of identified mutations on protein stability was verified in the TM-align (26). The functional annotation of the identified SNPs was performed using PROVEAN Protein (21).

### PCR analysis and Sanger sequencing

PCR (Platinum SuperFi II PCR Master Mix, Invitrogen) was performed on gDNA using primers listed in Table S2. The PCR products were run on a 1.5% agarose gel, stained with Midori Green and visualized under ultraviolet light. To confirm SNPs and INDELs, the PCR products were purified with Illustra ExoProStar 1-Step (Cytiva) and Sanger sequenced.

### Conclusions

The need for better TB control remains a global health priority. The live, attenuated BCG vaccine is already 100 years old and, despite being controversial, it is still the only vaccine available in TB immunoprophylaxis. The selection of the BCG substrain used for vaccine production remains an important issue. At present, it is difficult to determine which substrains will provide maximum efficacy and safety; therefore, more detailed genetic analyses of the BCG substrains are needed. The organization of the archival record of BCG distribution around the world and comparative studies of sister substrains are hampered by unclear nomenclature used initially, the use of synonyms, name changes due to a change in manufacturer or place of manufacture, or a change of substrain by the manufacturer. Despite that, over the 40 years of molecular studies of the BCG substrains, many markers have been identified that distinguish individual sister substrains – from SNP-type changes to large genomic rearrangements.

The whole genome sequence obtained is the first and only genomic sequence of the strain included in the TB vaccine production in Poland. It has been shown that it differs genetically from its closest sister strain BCG, derived from the same parent strain BCG-Moreau – *M. bovis* BCG Moreau RDJ strain. The results supplement the knowledge of the microevolution and attenuation of BCG vaccine substrains. The identified markers can be used to develop more specific identity and genetic stability tests of vaccine strains and taxonomic comparisons. The molecular characterization of BCG substrains opens the door to finding reliable correlates in the clinical assessment of the protective efficacy and safety of the BCG vaccine in humans, which is particularly important in the development of new TB vaccines and immunization strategies.

These results show the genetic stability of the Polish BCG strain over almost 60 years. Therefore, it can be concluded that the way, in which the vaccine strain had been stored and passaged in Poland did not lead to genetic changes, and the identified mutations occurred before 1957 in the BCG-Moreau genome.

## Data Availability

All information on the sequence of Mycobacterium tuberculosis variant bovis BCG strain Moreau PL is available in the NCBI database: CP085532.1. Reference sequences used for comparisons are available in the NCBI repository with the following identifiers: AM412059.2 [M. bovis BCG Moreau RDJ (19)] and AM408590.1 [M. bovis BCG Pasteur 1173P2 (18)]. Data supporting the findings of this study are available on request from the corresponding author K.K.-G.

## SUPPLEMENTAL MATERIAL

Table S1 spectrum.04259-23-s0001.xlsx Identified differences in the nucleotide sequence.

Table S2 spectrum.04259-23-s0002.docx Characterization of primers.

ASM does not own the copyrights to Supplemental Material that may be linked to, or accessed through, an article. The authors have granted ASM a non-exclusive, world-wide license to publish the Supplemental Material files. Please contact the corresponding author directly for reuse.
