# Supplementary material for: The whole genome sequence of Polish vaccine strain Mycobacterium bovis BCG Moreau
Source: Microbiol Spectr. 2024 May 17;12(7):e04259-23. doi: 10.1128/spectrum.04259-23 (PMC11237378; doi:10.1128/spectrum.04259-23)
Supplement: Table S2 — Characterization of primers. [file spectrum.04259-23-s0002.docx]

**Table S2** Characterization of primers used for Sanger sequencing

| **Primer** | **Primer sequence**  **5’ 🡪 3’** | **Primer position^a^** | **Amplicon size [bp]^b^** |
| --- | --- | --- | --- |
| 1RA-F | AGGAACGTTTCGACTTGGTG | 66 202 – 66 396 | 195/197 |
| 1RA-R | GATTTCCTCATCGGTGGTGT |  |  |
| 4RA-F | GCCTTGTCGTGGTCTAGTGA | 404 915 – 405 106 | 192 |
| 4RA-R | CAAGCGGATCAACCATGAAC |  |  |
| 6RA-F | ACCTTTGACGGCTACTGTGG | 555 443 – 555 632 | 190 |
| 6RA-R | TGCTACAGGCTACCGATGAA |  |  |
| 7RA-F | CGCGTCATAGAGGTGGATCT | 570 600 – 570 771 | 172 |
| 7RA-R | ACCCTGGGCAAACGGAGT |  |  |
| NJ1-2b-F | GCTGATGGTGTGCTTTGGT | 580 353 – 581 004 | 564/1919 |
| Igv5a-R | CGTCTTGAGCTCGTGACTGA |  |  |
| 8RA-F | GATGCAGGGTATGCAGGTTC | 692 962 – 693 115 | 154/153 |
| 8RA-R | CAACGGATTGGACTCGTCAT |  |  |
| 12RA-F | GCTGACAACCGCACTGTCTA | 878 244 – 878 450 | 207 |
| 12RA-R | GTAACGGACCTCCGAAGTGA |  |  |
| 14RA-F | GCCGTTAACCCCGACTCC | 930 939 – 931 194 | 256 |
| 14RA-R | CGATGCCCTCGGATTGAT |  |  |
| 17RA-F | ACCACCCGTACCACCCTTA | 1 191 347 – 1 191 942 | 596/821 |
| 17RA-R | CCGGTGGGTTGTTGTACG |  |  |
| 19RA-F | GATTGGCTCGGCGGTTAG | 1 217 408 – 1 217 591 | 184 |
| 19RA-R | AGTCAAGGTGCGCACAAAC |  |  |
| 20RA-F | CTGTTGGTATACCCGCCAGT | 1 315 931 – 1 316 175 | 245/246 |
| 20RA-R | TCGTAGGCCTGGGAAAGTTA |  |  |
| 21RA-F | ATCGAAACCGAAACGGGTAT | 1 322 566 – 1 322 722 | 157 |
| 21RA-R | CTCGGCTCGATCTCAAACTT |  |  |
| 23RA-F | GTCAGCCCGCTACGTGAC | 1 618 205 – 1 618 846 | 642 |
| 23RA-R | GTGGTGGTGGAACGGATTT |  |  |
| 24RA-F | ACTTCGGGCATATCGTCAAC | 1 730 926 – 1 731 141 | 216 |
| 24RA-R | GCGTGGGAAACTTGTCGTAG |  |  |
| 20MC-F | CGGTGCTCGAGTTGAAGTAG | 1 960 036 – 1 960 410 | 375/525 |
| 20MCa-R | CTATCACCACCCCGGAGTTC |  |  |
| 27RA-F | ACGTTCACGCTTACCGAGAC | 1 985 812 – 1 985 984 | 173 |
| 27RA-R | ATCAGGTCAAGGTGCTCCAC |  |  |
| 28RA-F | AGGGCCTGACCTACGACCT | 2 186 794 – 2 186 960 | 167 |
| 28RA-R | GGTGGCTACGTCCTGTGTCT |  |  |
| 2JC-F | CCGTAATGAATGCAACCTT | 2 205 539 – 2 215 633 | 10095/337 |
| 2JC-R | CTACTGGCCTTGATTCTCG |  |  |
| 3JC-F | GAACGCGAAAAACAGCAG | 2 215 889 – 2 216 153 | 265/165 |
| 3JC-R | GACGGTCGGCTTCTATGTGT |  |  |
| 30RA-F | GGTACGCACCAGCACCTC | 2 400 013 – 2 400 199 | 187 |
| 30RA-R | GGTGGTCGGTACACCCAAG |  |  |
| 31RA-F | GGCATCTTGATCGGCAAC | 2 650 878 – 2 651 477 | 600 |
| 31RA-R | ACCGCCGGAGATGACAAT |  |  |
| 32RA-F | CCGGCACATAAACCAGAAAT | 2 701 253 – 2 701 427 | 175 |
| 32RA-R | GGTGAAGTCAATTGGGATGC |  |  |
| 34RA-F | CCACTACCTAACGCCGAACT | 2 760 211 – 2 760 850 | 640 |
| 34RA-R | CTCACGGTTTCAATGCTGTG |  |  |
| 35RA-F | GAGGACACCGTTGTGTTTGG | 2 828 975 – 2 829 182 | 208 |
| 35RA-R | CACACCGTGGAACACCTACA |  |  |
| 36RA-F | ACCAAAGTGGTGGTGTCCAT | 2 866 763 – 2 866 965 | 203 |
| 36RA-R | ACCAATCCGGTGCTCAAG |  |  |
| 39RA-F | CGGTTCACCCACTTCGAC | 3 149 522 – 3 149 647 | 126 |
| 39RA-R | GTCGTTGGTGCAGACCAC |  |  |
| 40RA-F | GACACACGCTGGGATGTTC | 3 273 780 – 3 273 936 | 157 |
| 40RA-R | GAGGTCGATGTGCTGGTGT |  |  |
| 32MC-F | GGACGTGGTGTACGAGTGAA | 3 327 272 – 3 328 770 | 1499 |
| 32MC-R | GCTTCAGGGTTGTCGACTG |  |  |
| 41RA-F | ATATCGCGGATCTCCATGTC | 3 364 909 – 3 365 083 | 175 |
| 41RA-R | CCACCGAAGAACTCGAAGAA |  |  |
| 4JC-F | GACGATGTCCGCCTCGAT | 3 369 069 – 3369 260 | 192/260 |
| 4JC-R | GAGGTGACCCGCAATCAG |  |  |
| 42RA-F | ACCGCAGCCACTATTCGAT | 3 450 555 – 3 450 725 | 171 |
| 42RA-R | GCGGACTCGACAATGAACA |  |  |
| 48RAa-F | GACGGCCAAGGTCACATC | 3 704 715 – 3 705 079 | 365 |
| 48RAa-R | CTACCGGTACCGCCTTACAA |  |  |
| 50RA-F | GGGCAAAACACCGAGACC | 3 764 694 – 3 764 827 | 134 |
| 50RA-R | CCACCGCTGGAATTCTTC |  |  |
| 52RA-F | GACGTTCAACCGGTCAGC | 3 809 375 – 3 809 610 | 236 |
| 52RA-R | ATCGGCAATCACCTGACG |  |  |
| 53RA-F | CCCGGTGGTTTGATCTGG | 3 879 256 – 3 879 866 | 611/779 |
| 53RAa-R | ATCCCGCTGGCATTATCAG |  |  |
| 5JC-F | GTTGTAGGCGACGTTGGTAA | 3 880 324 – 3 881 379 | 1056/981 |
| 54RA-R | GCCGTTGAAATTGTTGTCG |  |  |
| 54RAa-F | GCTTTGACGGCGGCCAAG | 3 881 078 – 3 881 686 | 609/1074 |
| 54RAa-R | CAAAGCCGGAGAGGTCCAG |  |  |
| NJ7a-F | GCGGCGAGGGAGCCAGCGGT | 3 881 652 – 3 883 104 | 1453/2647 |
| NJ7a -R | GCCGGCTACGCCGCTGCCGAA |  |  |
| 55RAc-F | CGCGATGGGTAGCACTGG | 3 888 077 – 3 889 024 | 948/1293 |
| 55RAc-R | CCCTTACCCCCATCGCCG |  |  |
| 55RA-F | AGCAGATAACACCGCAAACA | 3 891 095 – 3 891 879 | 785 |
| 55RA-R | GGTTTGTCGACGAGGTGAAG |  |  |
| 55RAa-F | GTCCAGCCAGATTTGACGAC | 3 891 682 – 3 892 352 | 671 |
| 55RAa-R | TAATGCCGCAACGAACCTG |  |  |
| 56RA-F | CGTCATCGCCTCCCATAC | 3 962 896 – 3 963 059 | 164 |
| 56RA-R | AGCTTGCCGGCTACGTACT |  |  |
| 57RA-F | ATGCCGTTAGACGACGTTTC | 4 000 636 – 4 000 829 | 194/195 |
| 57RA-R | CAGGGTTCCCAAGGTATGG |  |  |
| 58RA-F | ATCGACCATCATCACCCAGT | 4 172 195 – 4 172 399 | 205 |
| 58RA-R | GTTCAGCGACGACATCTACG |  |  |
| 59RA-F | CTGGTGAACAGCGCTAAGG | 4 248 924 – 4 249 090 | 167/166 |
| 59RA-R | GAGTGCCTGGTGGGGTACT |  |  |
| 60RA-F | CCCTGGGTGATGAAGAGGC | 4 299 921 – 4 300 231 | 311/1187 |
| 60RA-R | GCGGTCAACATTTGCTACGA |  |  |

a – primer position in the genome of BCG-Moreau RDJ (GenBank: AM412059.2)

b – PCR product size for BCG-Moreau RDJ and BCG-Moreau PL or BCG-Moreau RDJ/BCG-Moreau PL
